# Big Data Management for Healthcare Systems: Architecture, Requirements, and Implementation

**DOI:** 10.1155/2018/4059018

**Published:** 2018-06-21

**Authors:** Naoual El aboudi, Laila Benhlima

**Affiliations:** Department of computer sciencne, Mohammadia School of Engineering, Mohammed V University, Rabat, Morocco

## Abstract

The growing amount of data in healthcare industry has made inevitable the adoption of big data techniques in order to improve the quality of healthcare delivery. Despite the integration of big data processing approaches and platforms in existing data management architectures for healthcare systems, these architectures face difficulties in preventing emergency cases. The main contribution of this paper is proposing an extensible big data architecture based on both stream computing and batch computing in order to enhance further the reliability of healthcare systems by generating real-time alerts and making accurate predictions on patient health condition. Based on the proposed architecture, a prototype implementation has been built for healthcare systems in order to generate real-time alerts. The suggested prototype is based on spark and MongoDB tools.

## 1. Introduction

The proportion of elderly people in society is growing worldwide [1]; this phenomenon known as humanity's aging has many implications on healthcare services, especially in terms of costs. In the face of such situation, relying on classical systems may result in a life quality decline for millions of people. Seeking to overcome this problem, a bunch of healthcare systems have been designed. Their common principle is transferring, on a periodical basis, medical parameters like blood pressure, heart rate, glucose level, body temperature, and ECG signals to an automated system aimed at monitoring in real time patients health condition. Such systems provide quick assistance when needed since data is analyzed continuously. Automating health monitoring favors a proactive approach that relieves medical facilities by saving costs related to hospitalization, and it also enhances healthcare services by improving waiting time for consultations. Recently, the number of data sources in healthcare industry has grown rapidly as a result of widespread use of mobile and wearable sensors technologies, which has flooded healthcare area with a huge amount of data. Therefore, it becomes challenging to perform healthcare data analysis based on traditional methods which are unfit to handle the high volume of diversified medical data. In general, healthcare domain has four categories of analytics: descriptive, diagnostic, predictive, and prescriptive analytics; a brief description of each one of them is given below.

*Descriptive Analytics*. It consists of describing current situations and reporting on them.

Several techniques are employed to perform this level of analytics. For instance, descriptive statistics tools like histograms and charts are among the techniques used in descriptive analytics. 

*Diagnostic Analysis.* It aims to explain why certain events occurred and what the factors that triggered them are. For example, diagnostic analysis attempts to understand the reasons behind the regular readmission of some patients by using several methods such as clustering and decision trees. 

*Predictive Analytics*. It reflects the ability to predict future events; it also helps in identifying trends and determining probabilities of uncertain outcomes. An illustration of its role is to predict whether a patient can get complications or not. Predictive models are often built using machine learning techniques. 

**Figure 1:**
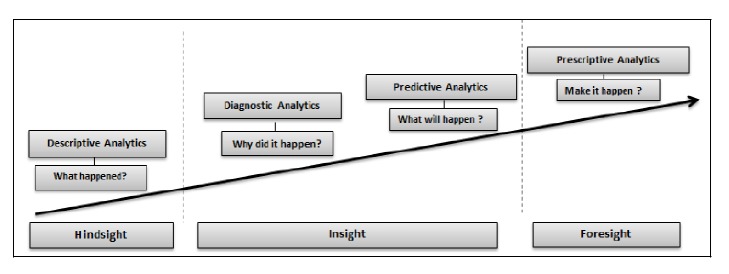
Analytics for healthcare domain.

*Prescriptive Analytics*. Its goal is to propose suitable actions leading to optimal decision-making. For instance, prescriptive analysis may suggest rejecting a given treatment in the case of a harming side effect high probability. Decision trees and monte carlo simulation are examples of methods applied to perform prescriptive analytics.  Figure 1 illustrates analytics phases for healthcare domain [2]. The integration of big data technologies in healthcare analytics may lead to better performance of medical systems.

In fact, big data refers to large datasets that combine the following characteristics (see [3]): volume which refers to high amounts of data, velocity which means that data is generated at a rapid pace, variety which emphasizes that data comes under different formats, and, finally, veracity which means that data originates from trustable sources.

Another characteristic of big data is the variability. It indicates variations that occur in the data flow rates. Indeed, velocity does not provide a consistent description of the data due to its periodic peaks and troughs. Another important aspect of big data is complexity; it arises from the fact that big data is often produced through a bunch of sources, which implies, to perform many operations over the data, these operations include identifying relationships and cleansing and transforming data flowing from different origins.

Moreover, Oracle decided to introduce value as a key attribute of big data. According to Oracle, big data has a “low value density,” which means that raw data has a low value compared to its high volume. Nevertheless, analysis of important volumes of data may lead to obtaining a high value.

In the context of healthcare, high volumes of data are generated by multiple medical sources, and it includes, for example, biomedical images, lab test reports, physician written notes, and health condition parameters allowing real-time patient health monitoring. In addition to its huge volume and its diversity, healthcare data flows at high speed. As a result, big data approaches offer tremendous opportunities regarding healthcare systems efficiency.

The contribution of this research paper is to propose an extensible big data architecture for healthcare applications formed by several components capable of storing, processing, and analyzing the high amount of data in real time and batch modes. This paper demonstrates the potential of using big data analytics in the healthcare domain to find useful information in highly valuable data.

The paper has been organized as follows: In Section 2, a background of big data computing approaches and big data platforms is provided. Recent contributions on big data for healthcare systems are reviewed in Section 3. In Section 4, the components of the proposed big data architecture for healthcare are described. The implementation process is reported in Section 5. Conclusion is finally drawn in Section 6, along with recommendations for future research.

## 2. Background

### 2.1. An Overview of Big Data Approaches

Big data technologies have received great attention due to their successful handling of high volume data compared to traditional approaches. Big data framework supports all kind of data, structured, semistructured, and unstructured data, while providing several features. Those features include predictive model design and big data mining tools that allow better decision-making process through the selection of relevant information.

Big data processing can be performed through two manners: batch processing and stream processing; see [4]. The first method is based on analyzing data over a specified period of time; it is adopted when there are no constraints regarding the response time. On the other hand, stream processing is suitable for applications requiring real-time feedback. Batch processing aims to process a high volume of data by collecting and storing batches to be analyzed in order to generate results.

Batch mode requires ingesting all data before processing it in a specified time. Mapreduce represents a widely adopted solution in the field of batch computing; see [5]; it operates by splitting data into small pieces that are distributed to multiple nodes in order to obtain intermediate results. Once data processing by nodes is terminated, outcomes will be aggregated in order to generate the final results. Seeking to optimize computational resources use, mapreduce allocates processing tasks to nodes close to data location. This model has encountered a lot of success in many applications, especially in the field of bioinformatics and healthcare. Batch processing framework has many characteristics such as the ability to access all data and to perform many complex computation operations, and its latency is measured by minutes or more.

*Stream Computing.* In real applications such as healthcare, intelligent transportation, and finance, a high amount of data is produced in continuous manner. When the need of processing such data streams in real time arises, data analysis takes into consideration continuous evolution of data and permanent change regarding statistical characteristics of data streams referred to as concept drift; see [6]. Indeed, storing a large amount of data for further processing may be challenging in terms of memory resources. Moreover, real applications tend to produce noisy data which contain missing values along with redundant features, making by the way data analysis complicated, as it requires important computational time. Stream processing reduces this computational burden by performing simple and fast computations for one data element or for a window of recent data, and such computations spend seconds at most.

Big data stream mining methods including classification, frequent pattern mining, and clustering relieve computational effort through rapid extraction of the most relevant information; this objective is often achieved by mining data in a distributed manner. Those methods belong to one of the two following classes: data-based techniques and task-based techniques; see [7]. Data-based techniques allow summarizing the entire dataset or selecting a subset of the continuous flow of streaming data to be processed. Sampling is one of these techniques; it consists of choosing a small subset of data to be processed according to a statistical criterion. Another data-based method is load shedding which drops a part from the entire data, while sketching technique establishes a random projection on a feature set. Synopsis data structures method and aggregation method belong also to the family of data-based techniques, the first one summarizes data streams, and the second one represents a number of elements in one element by using a statistical measure.

Task-based techniques update existing methods or design new ones to reduce the computational time in the case of data stream processing. They are categorized into approximation algorithms that generate outputs with acceptable error margin, sliding window that analyzes recent data under the assumption that it is more useful than older data, and algorithm output granularity that processes data according to the available memory and time constraints.

Big data approaches are essential for modern healthcare analytics; they allow real-time extraction of relevant information from a large amount of patient data. As a result, alerts are generated when the prediction model detects possible complications. This process helps to prevent health emergencies from occurring; it also assists medical care professionals in decision-making regarding disease diagnosis and provides special care recommendations.

### 2.2. Big Data Processing Frameworks

Concerning batch processing mode, mapreduce framework is widely adopted; it allows distributed analysis of big data on a cluster of machines. Thus, simple computations are performed through two functions that consist of map and reduce. Mapreduce relies on a master/slave architecture, the master node allocates processing tasks to slave nodes and divides data into blocks, and, then, it structures data into a set of keys/values as an input of map tasks. Each worker assigns a map task to slaves and reads the appropriate input data, and, after that, it writes generated results of the map task into intermediate files. The reducer worker transmits results generated by the map task as an input of the reducer task; finally, the results are written into final output files. Hadoop is an open source framework that stores and analyzes data in a parallel manner through clusters.

It is composed of two main components: Hadoop mapreduce and distributed file system. Distributed file system (HDFS) stores data by duplicating it in many nodes; on the other hand, hadoop mapreduce implements mapreduce programming model, its master node stores metadata information such as locations of duplicated blocks, and it identifies locations of data nodes to recover missing blocks in failure cases. The data are splitted into several blocks and the processing operations are made in the same machine. With hadoop, other tools regarding data storage can be used instead of HDFS, such as HBase, Cassandra, and relational databases. Data warehousing may be performed by other tools, for instance, Pig and Hive, while mahout is employed for machine learning purposes. When stream processing is required, Hadoop may not be a suitable choice since all input data must be available before starting mapreduce tasks. Recently, Storm from Twitter, S4 from Yahoo, and spark were presented to process incoming stream data. Each solution has its own principle.

*Storm*. It is an open source framework to analyze data in real time, see [8], it is formed by Spouts and Bolts. Spout can produce data or load data from an input queue and bolt processes input streams and generates outputs streams. In storm program, a combination of a bolt and a spout is named topology. Storm has three nodes that are the master node named nimbus, the worker node and zookeeper. The master node distributes and coordinates the execution of topology while the worker node is responsible for executing spouts/bolts. Finally, zookeeper synchronizes distributed coordination. 

*S4*. It is a distributed stream processing engine, inspired by the mapreduce model in order to process data streams; see [9]. It was implemented by Yahoo through Java. Data streams feed to S4 as events. 

*Spark*. It is applied for both batch and stream processing; therefore, spark may be considered as a powerful framework compared with other tools such as hadoop and storm; see [10]. It can access several data sources like HDFS, Cassandra, and HBase. Spark provides several interesting features, for example, iterative machine learning algorithms through Mllib library which provides efficient algorithms with high speed, structured data analysis using Hive, and graph processing based on GraphX and SparkSQL that restore data from many sources and manipulate them using SQL languages. Before processing data streams, spark divides them into small portions and transforms them into a set of RDDs (Resilient Distributed Datasets) named DStream (Discretised Stream). 

*Apache Flink*. It is an open source solution that analyzes data in both batch and real-time mode [11]. The programming models of flink and mapreduce share many similarities. It allows iterative processing and real-time computation on stream data collected by tools such as flume and KAFKA. Apache flink provides several features like FlinkML which represents a machine learning library capable of providing many learning algorithms for fast and scalable big data applications. 

*MongoDB.* It is a NoSQL database capable of storing a high amount of data. MongoDB relies on JSON standard (Java Script Object Notation) in order to store records; it consists of an open, human, and machine-readable format that makes data interchange easier compared to classical formats such as rows and tables. In addition, JSON scales better since join based queries are not needed due to the fact that relevant data of a given record is contained in a single JSON document.. Spark is easily integrated with MongoDB; see [12].

Table 1 summarizes big data processing solutions.

## 3. Big Data-Based Healthcare Systems

The potential offered by big data approaches in healthcare analytics has attracted the attention of many researchers. In [13], recent advances in big data for health informatics and their role to tackle disease management are presented, for instance, diagnosis prevention and treatment of several illnesses. The study demonstrates that data privacy and security represent challenging issues in healthcare systems.

Raghupathi et al. exposed in [14] the architectural framework and challenges of big data healthcare analytics. In another study (see [15]), the importance of security and privacy issues is demonstrated in implementing successfully big data healthcare systems. Belle et al. discuss in [16] the role of big data in improving the quality of care delivery by aggregating and processing the large volume of data generated by healthcare systems. In [17], data mining techniques for healthcare analytics are presented, especially those used in healthcare applications like survival analysis and patient similarity. Bochicchio et al. proposed in [18] a big data healthcare analytics framework for supporting multidimensional mining over big healthcare data. The objective of this framework is analyzing the huge volume of data by applying data mining methods. Sakr et al. presented in [19] a composite big data healthcare analytics framework, called Smarthealth, whose goal is to overcome the challenges raised by healthcare big data via ICT technologies. In [20], authors presented Wiki-Health, a big data platform that processes data produced by health sensors. This platform is formed by the three following layers: application, query and analysis, and data storage. Application Layer ensures data access, data collection, security, and data sharing. On the other hand, query and analysis layer provides data management and data analysis, while data storage layer is in charge of storing data as its name suggests. Challenges regarding the design of such platforms, especially in terms of data privacy and data security, are highlighted in [21]. Baldominos et al. designed in [22] an intelligent big data healthcare management solution aimed at retrieving and aggregating data and predicting future values.

Based on big data technologies, a few data processing systems for healthcare domain have been designed in order to handle the important amount of data streams generated by medical devices; a brief description of the major ones is provided in the next section.

Borealis-based Heart Rate Variability Monitor, presented in [23], belongs to the category of big data processing systems for healthcare systems; it processes data originating from various sources in order to perform desired monitoring activities. It is composed of stream transmitter that represents an interface between sensors collecting data and Borealis application; it encapsulates the collected data into Borealis format in order to obtain a single stream. Then, the final stream is transferred toward Borealis application for processing purposes. This system includes also a graphical user interface (GUI) that allows physicians to select from among patients those whose health condition is going to be the subject of close monitoring. Moreover, the graphical interface permits the medical staff to choose the parameters they want to focus on, regarding a monitoring task. Furthermore, it allows visualization of Borealis application outcomes. The system has many drawbacks; for instance, it does not include a machine learning component capable of making accurate predictions on patient health condition. Furthermore, adding an alarming component would enhance emergency cases detection.

Hadoop-based medical emergency management system using IoT technology relies on sensors measuring medical parameters through different processes [24]. Those sensors may be devices mounted on patient body or other types of medical devices capable of providing remote measuring. Before being transferred to the component called intelligent building (IB), the collected data flows through the primary medical device (PMD). Next, IB starts by aggregating the input stream thanks to its collection unit; then, the resulting data is transferred to Hadoop Processing Unit (HPU) to perform statistical analyses of parameters measured by sensors based on mapreduce paradigm. The map function aims to verify sensor readings; this verification occurs by performing a comparison with their corresponding normal threshold. If readings are considered to be normal, they are stored in database without further processing. On the other hand, if they are alarming, an alert is triggered and transmitted to the application layer. Meanwhile, when sensors return values that are neither normal, nor alerting, it is necessary to analyze them closely. Results of such analysis are collected by aggregation result unit through a reducer from different data nodes; then, they are sent to the final decision server. Finally, decision server receives the current results and applies machine learning classifiers and medical expert knowledge to process past patient data for more accurate decisions and generates outputs based on Hadoop Processing Unit results. This system is based on hadoop ecosystem which is adapted for batch processing, however, it does not support stream processing. Therefore, it is more recommended to use spark in order to improve the system performance in terms of processing time using data stream mining approaches.

A Prototype of Healthcare Big Data Processing System based on Spark [25] is proposed to analyze the high amount of data generated by healthcare big data process systems. It is formed by two logical parts: big data application service and big data supporting platform performing data analysis. The first logical part visualizes the processing results and plays the role of an interface between applications and data warehouse big data tools such as Hive or Spark SQL. The second one is responsible for computing operations and distributed storage allowing high storage capabilities. This solution is based on spark which is very promising since it handles batch computing, stream computing, and ad hoc query. The system has many drawbacks; for instance, it does not include big data mining and big data analytics in experimental platform, which hampers prediction possibilities that are vital for improving the quality of patient outcomes.

In this paper, we summarize the added value of big data technologies on healthcare analytics by presenting an extensible big data architecture for healthcare analytics that combines advantages of both batch and stream computing to generate real-time alerts and make accurate predictions about patient health condition. In this research, an architecture for management and analysis of medical data was designed based on big data methods and can be implemented via a combination of several big data technologies. Designing systems capable of handling both batch and real-time processing is a complex task and requires an effective conceptual architecture for implementing the system.

## 4. An Extensible Big Data Architecture for Healthcare

We are developing a system that has the advantage to be generic and can deal with various situations such as early disease diagnosis and emergency detection. In this study, we propose a new architecture aimed at handling medical big data originating from heterogeneous sources in different formats. Data management in this architecture is illustrated through the following scenario.

Indeed, new medical data is sent simultaneously to both batch layer and streaming layer. In batch mode, data is stored in data nodes; then, it is transmitted to semantic module which affects meaning to data using ontology store; after that, cleaning and filtering operations are applied to the resulting data before processing it. In the next step, the prepared data is analyzed through different phases: feature selection and feature extraction. Finally, the prepared data is used to design models predicting patients future health condition. This mode is solicited periodically on an offline basis. In the stream scenario, data comes from multiple sources such as medical sensors connected to patient body, measuring several medical parameters like blood pressure. Then, the collected data is synchronized based on time and its missing values are handled.

**Figure 2:**
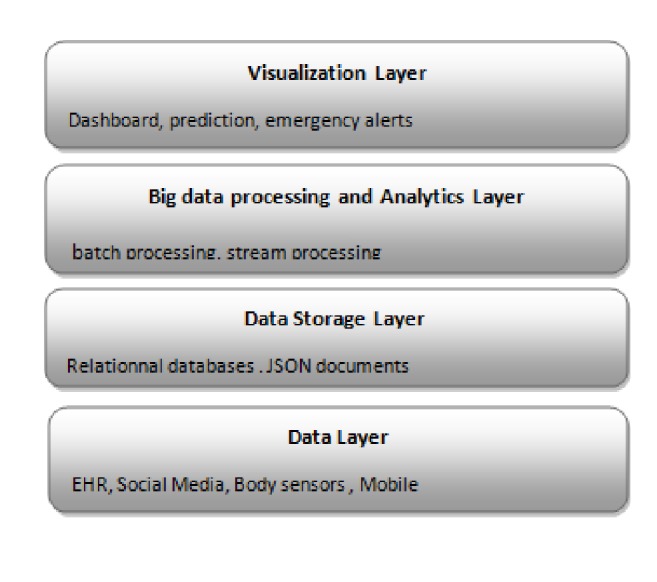
The layer architecture.

Based on sliding window technique, the adaptive preprocessor splits data into blocks, and then it extracts relevant information for the predictor component in order to build a predictive model for every window tuple.  Figure 2 represents the layer architecture of the proposal.

### 4.1. Batch Processing Layer

Batch computing is performed on extracted data from prepared data store through different phases.

#### 4.1.1. Data Acquisition

When monitoring continuously a patient health condition, several types of data are generated. Medical data may include structured data like traditional Electronic Healthcare Records (EHRs), semistructured data such as logs produced by some medical devices, and unstructured data generated, for example, by biomedical imagery. 

*Electronic Healthcare Records HER.* It contains a complete patient medical history stored in a digital format; it is formed by a multitude of medical data describing the patient's health status like demographics, medications, diagnoses, laboratory tests, doctor's note, radiology documents, clinical information, and payment notes. Thus, EHR represents a valuable source of information for the purpose of healthcare analytics. Furthermore, EHR allows exchanging data between healthcare professionals community. 

*Biomedical Images*. Biomedical imaging is considered as a powerful tool regarding disease detection and care delivery. Nevertheless, processing this kind of images is challenging as they include noisy data that needs to be discarded in order to help physicians make accurate decisions. 

*Social Network Analysis.* Performing social network analysis requires gathering data from social media like social networking sites. The next step consists of extracting knowledge that could affect healthcare predictive analysis such as discovering infectious illnesses. In general, social networks data is marked by uncertainty that makes their use in designing predictive models risky. 

*Sensing Data.* Sensors of different types are employed in healthcare monitoring solutions. Those devices are essential in monitoring a patient health as they measure a wide range of medical indicators such as body temperature, blood pressure, respiratory rate, heart rate, and cardiovascular status. In order to ensure an efficient health monitoring, patients living area may be full of devices like surveillance cameras, microphones, and pressure sensors. Consequently, data volume generated by health monitoring systems tends to increase tremendously which requires adopting sophisticated methods during the processing phase. 

*Mobile Phone*. Nowadays, mobile phone represents one of the most popular technological devices in the world. Compared to their early beginnings, mobile phones transformed from a basic communication tool to a complex device offering many features and services. They are currently equipped with several sensors like satellite positioning services, accelerometers, and cameras. Due to their multiple capabilities and wide use, mobile phones are ideal candidates regarding health data collection allowing the design of many successful healthcare applications like pregnancy monitoring [26], child nutrition [27], and heart frequency monitoring [28].

The objective of data acquisition phase is to read the data gathered from healthcare sensors in several formats and then data flows through semantic module before being normalized. 

*Semantic Module*. It is based on ontologies, which constitute efficient tools when it comes to representing actionable knowledge in the field of biomedicine. In fact, ontologies have the ability to extract biomedical knowledge in a formal, powerful, and incremental way. They also allow automation and interoperability between different clinical information systems. Automation has a major benefit; it helps medical personnel in processing large amounts of patients' data, especially when taking into consideration that this personnel is often overwhelmed by a series of healthcare tasks. Introducing automation in healthcare application contributes to providing assistance to human medical staff, which enhances its overall performance. It should be highlighted that automation will help humans in performing their duties rather than replacing them. Interoperability is an important issue when dealing with medical data. In fact, healthcare databases lack homogeneity as they adopt different structures and terminologies. Therefore, it is difficult to share information and integrate healthcare data. In this context, ontologies may play a determinant role by establishing a common structure and semantics, which allows sharing and reuse of data across different systems. In other words, by defining a standard ontology format, it becomes possible to map heterogeneous databases into a common structure and terminology. For instance, the Web Ontology Language (OWL) represents the standard interchange format regarding ontology data that employs XML syntax.

#### 4.1.2. Data Preparation

Processing raw data without preparation routines may require extra computational resources that are not affordable in big data context. Thus, it is recommended to make sure data is prepared properly, in order to obtain accurate predictive models and to enhance the reliability of data mining techniques. Data preparation consists of two steps: data cleaning and data filtering. 

*Data Filtering*. Data filtering in the presence of large size data is achieved by discarding information that is not useful for healthcare monitoring based on a defined criterion. 

*Data Cleaning*. It encompasses several components such as normalization, noise reduction, and missing data management.

Several methods are utilized in order to eliminate noisy data and to find out the values of missing data. In fact, medical records often include noisy information and may have missing data. Determining missing values in healthcare data is a critical process. Making errors in filling missing values may affect the quality of extracted knowledge and lead to incorrect results. In healthcare domain, the handling of missing data should be performed with maximum precision as wrong decisions may have serious consequences. Data mining field has many powerful algorithms aimed at handling missing values, for instance, Expectation-Maximization (EM) algorithm and multiple Imputation algorithm.

*Noise Treatment.* In general, noisy data is treated according to two main approaches. The first one consists of correcting noisy values based on data polishing techniques; these techniques are difficult to implement and are applied only in the case of small amounts of noise. The second approach is based on noise filters, which determine and eliminate noisy instances in the training data, and those filters do not introduce modifications on adopted data mining methods.

For instance, electronic medical records (EMRs) illustrate well the need for data cleaning as it may provide noisy data containing incomplete information. Data sparsity in EMRs finds its origin in irregular collection of parameters over time, since patient parameters are recorded only when patients are present in hospitals. In the case of biomedical imagery, many processing techniques have been applied in order to reduce noise.

Generally, the preparation of biomedical images starts by the identification (segmentation) of significant objects. On the other hand, data preparation is more challenging when dealing with raw social media data. In addition to its huge volume and its informal content, this kind of data has the critical aspect of including user's personal information. Thus, data cleaning is a key factor for success in social networks analysis. When data preparation step ends, the processed data needs to be stored in prepared data store.

#### 4.1.3. Feature Extraction and Feature Selection

The proliferation of devices designed to collect medical data in recent years has increased tremendously both the number of features and instances in the field of healthcare monitoring. Therefore, selecting the most significant features becomes crucial when facing such high volume data; see [29]. In this context, several techniques have been proposed to manage this issue, especially when handling thousands of features [29]. On the other hand, feature extraction represents another approach that consists of extracting a reduced number of attributes compared to the original ones. Applying feature selection and extraction methods requires a statistical tools store. When this phase terminates, the selected feature subset will be used to build the predictive model.

#### 4.1.4. Predictive Model Design

The objective of this component is to build a model capable of producing predictions for new observations based on the previous data. The quality of a given predictive model is evaluated by its accuracy. Those models are developed based on tools available in the statistical and machine learning store provided by the suggested architecture. The results of batch processing will be stored into model store.

### 4.2. Data Storage

Data Storage is one of the most challenging tasks in big data framework, especially in the case of healthcare monitoring systems which involve large amounts of data. Therefore, traditional data analysis is unfit to manage those systems. This component may be HDFS, NoSQL such as MongoDB and SQL databases, or a combination of all of them. Therefore, it is more scalable and ensures high storage capabilities. In the proposed system, the patient data collected from heterogeneous sources can be classified into structured data such as EHR, unstructured data like biomedical images, or semistructured data such as XML and JSON documents. These data will be stored into raw data store in the target databases. Streaming data such as social media will be stored into stream temp data store.

### 4.3. Stream Processing Layer

Stream data analysis layer is composed of data synchronization module, adaptive preprocessor module, and adaptive predictor module. 

*Data Synchronization.* The role of data synchronization module is to make sure that data is processed in the correct order regarding time criterion. In addition, data synchronization process dismisses measurements that are inconsistent and takes care of missing values. Detection of inconsistent values is performed by defining thresholds on the incoming parameters. 

*Adaptive Learning.* In many applications, it is assumed that data preprocessing task is performed by learning algorithms, or at least that data has been already preprocessed before its arrival. In the majority of cases, such expectations do not match reality. This is particularly true for our proposed system which extracts streaming data from stream temp store. The need to adapt in the face of data changes led to the development of adaptive systems, and a key factor for the long term success of a big data system is adaptability. In fact, preprocessing is not a task performed in an independent manner; it is rather a component belonging to the adaptive system. Moreover, in order to stay reliable and maintain a certain degree of accuracy, predictive models are supposed to adapt when data changes occur. As a result, prediction process may be considered as a part of the adaptive system that will be the association of two distinct parts that are adaptive preprocessor and adaptive predictor. 

*Adaptive Preprocessor*. It starts processing operations by splitting the arriving data flow in time windows. In this context, sliding window technique is adopted in order to split data streams into overlapping subsets of tuples (every tuple is included in multiple windows). For a given window, the average of every measure is computed and compared to predefined user's threshold. If the value of a particular average exceeds alarming threshold value, it will be stored while an emergency alert is generated; otherwise, it is simply stored. When comparisons with threshold values are terminated, information extraction phase proceeds by selecting relevant features which will be transmitted to adaptive predictor component; see [30]. 

*Adaptive Predictor*. In order to maintain a certain level of accuracy, predictors have to update according to data changes. Otherwise, they will simply become less reliable through time due to data evolution. Therefore, predictive model should take into consideration newly arrived samples, while having, at the same moment, the ability to generate predictions on a real-time space.

The adaptive feature requires the establishment of a connection between adaptive processor and adaptive predictor. Through that connection, the predictor sends feedback to the preprocessor regarding the need to update or not, and then the preprocessing unit will provide it when necessary with raw data via a given mapping function. The results of stream processing will be transferred into stream processing results store.

### 4.4. Query Processor

The query processor aims to find the status of patients by combining the responses of queries sent to both the stream processing results store and the batch processing results store.

### 4.5. Visualization Layer

The analytics layer produces multiple outputs that include, for instance, visualization of patient health monitoring report and predictive decision report. In healthcare context, the priority in terms of real-time visualization is given to the most critical information in order to optimize decision-making and avoid emergencies. Examples of relevant information encompass patient dashboards tracking daily health condition, real-time alerts, and proactive messages generated by predictions.

**Figure 3:**
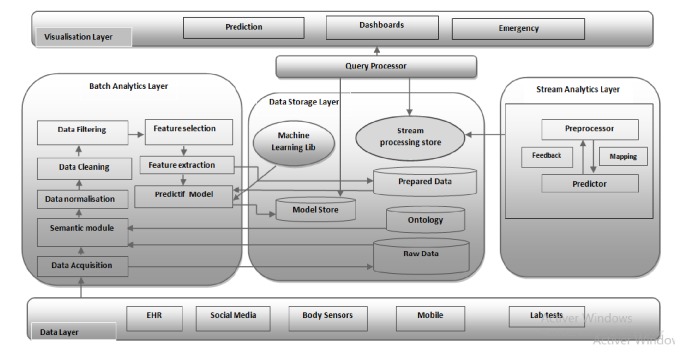
Big data architecture for healthcare systems.

Figure 3 shows the proposed big data architecture for healthcare.

## 5. Implementation Process for Detecting Emergency Cases

**Figure 4:**
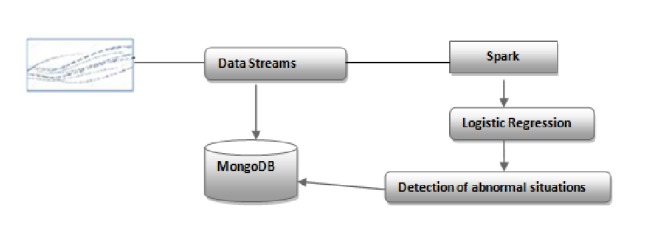
The implementation process of our proposal.

In this prototype system, we aim to detect potential dangers of patients. Spark streaming and MongoDB are chosen to implement the module of emergency detection figuring in the visualization layer of the proposed architecture. The system employs spark to read data from MongoDB in the batch layer. The batch jobs run at a regular time interval specified by the user. Spark streaming is used for processing real-time data streams; it directly gets data from medical sources and detects abnormal situations based on user's thresholds. Then, spark streaming sends alerts to MongoDB which will be used to notify doctors about emergencies. Spark MLLib and spark streaming techniques are adopted for real-time monitoring and online learning to predict whether the current state of patients is danger or not which is the supervised classification. The logistic regression model is selected for handling this supervised classification problem.  Figure 4 illustrates the implementation process of our proposal.

### 5.1. Diabetic Patient Case Study

**Figure 5:**
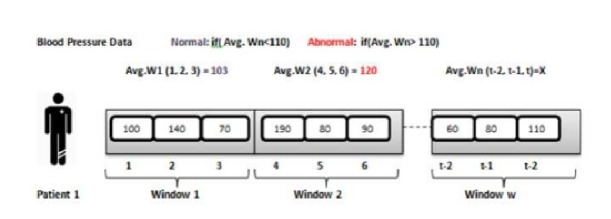
Real-time monitoring of the blood pressure parameter.

**Figure 6:**
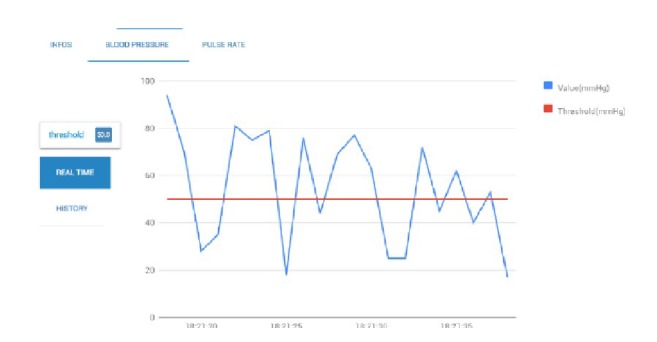
Visualization of measured patient parameters.

Chronic patients must pay attention to numerous aspects in their daily life. Diet, sport activity, medical analysis, or blood glucose levels represent some of those aspects. Medical care of such patients is a challenging process since a lot of checks are performed many times during a single day; for instance, some diabetics measure their blood pressure several times on a daily basis. The objective of the proposed system is to allow doctors to perform a real-time monitoring of diabetic patient's health condition. First, the real-time alert detection reads directly from all the incoming data streams provided by sensors reading; then, for every window data stream, healthcare measures are compared with user defined thresholds in order to decide whether the current parameters are abnormal through mapreduce jobs. In the following step, the average value of every medical measure is calculated and written into MongoDB for notification purposes.  Figure 5 illustrates a real-time monitoring of the blood pressure parameter and Figure 6 visualizes patient measures.

The effectiveness of the proposal is evaluated by conducting experiments with a cluster formed by 3 nodes with identical setting, configured with an Intel CORE™ i7-4770 processor (3.40GHZ, 4 Cores, 16GB RAM, running Ubuntu 12.04 LTS with 64-bit Linux 3.11.0 kernel).  Figure 5 illustrates the visualization of patient medical parameters measured by a given sensor selected by user through a GUI designed for that purpose.

To evaluate the scalability of the proposal, we used an Open Source EHR Generator such as Box 1 to produce medical patient data in HL7 FHIR format which are loaded into MongoDB as a JSON documents such as Box 1.

## 6. Conclusion

In this paper, popular healthcare monitoring systems based on big data have been reviewed. Meanwhile, an overview of recent big data processing approaches and technologies has been provided. Then, a big data processing architecture for healthcare industry has been presented; it is capable of handling the high amount of data generated by different medical sources in real time. The proposal is designed according to big data approaches. The main contribution of the proposed solution is twofold; first, it proposed a generic big data architecture for healthcare based on both batch computing and stream computing providing simultaneously accurate predictions and online patient dashboards. Then, a solution prototype implementation based on spark and MongoDB has been proposed, in order to detect alarming cases and generate real-time alerts. In the future works, we project to handle missing value through Expectation-Maximization (EM) algorithm and we will implement the semantic module.

## Figures and Tables

**Box 1:**
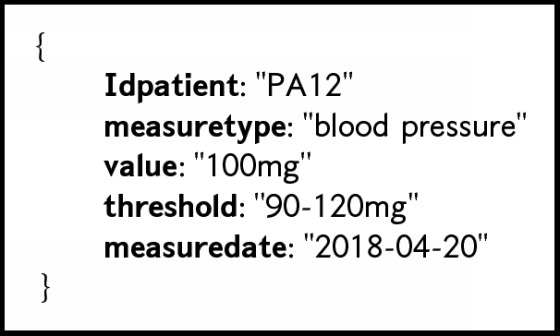
JSON document representing patient parameters into MongoDB.

**Table 1:** Big data processing solutions.

**Framework**	**Type**	**Latency**	**Developped by**	**Stream Primitive**	**Stream source**
**Hadoop**	batch	Minutes or more	Yahoo	Key-value	HDFS
**Storm**	streaming	Subseconds	Twitter	Tuples	Spouts
**Spark streaming**	Batch/streaming	Few seconds	Berkley AMPLay	DStream	HDFS
**S4**	streaming	Few seconds	Yahoo	Events	Networks
**Flink**	Batch/streaming	Few seconds	Apache Software Foundation	key-value	KAFKA
